# A Rare Case of Achondroplasia With Bilateral Developmental Cataract

**DOI:** 10.7759/cureus.34191

**Published:** 2023-01-25

**Authors:** Rasika Bagewadi, Pravin Tidake, Sandhya Jeria, Mayur B Wanjari

**Affiliations:** 1 Department of Ophthalmology, Jawaharlal Nehru Medical College, Datta Meghe Institute of Higher Education and Research, Wardha, IND; 2 Department of Research and Development, Jawaharlal Nehru Medical College, Datta Meghe Institute of Higher Education and Research, Wardha, IND

**Keywords:** genetic syndromes, cataract, developmental cataract, posterior polar cataract, achondroplasia

## Abstract

Enchondral ossification disorders of autosomal dominant congenital origin include achondroplasia, among others. Low stature, craniofacial deformity, and spinal abnormality are its clinical hallmarks. Some of the associated ocular characteristics are telecanthus, exotropia, angle abnormalities, and cone-rod dystrophy. A 25-year-old female presented to the Ophthalmology outpatient department (OPD) with the classical clinical signs of achondroplasia and developmental cataracts in both eyes. She also had associated esotropia in the left eye. Achondroplasia patients should be screened for developmental cataracts to enable timely intervention and management.

## Introduction

Achondroplasia is an autosomal dominant congenital disorder of enchondral ossification [1]. A mutation in the fibroblast growth factor receptor 3 (FGFR3) gene causes dysplasia of enchondral formation, which leads to the development of achondroplasia [2]. Clinical signs of the disorder include low height, an embossed frontal bone, and cranial-facial and vertebral malformation [3]. Simple microphthalmos [4], telecanthus, exotropia, inferior oblique overaction, angle anomalies, Duane retraction syndrome, cone-rod dystrophy, and chorioretinal coloboma are among the reported ocular anomalies associated with achondroplasia. We present a case of achondroplasia and bilateral developmental cataract with esotropia in the left eye.

## Case presentation

**Figure 1:**
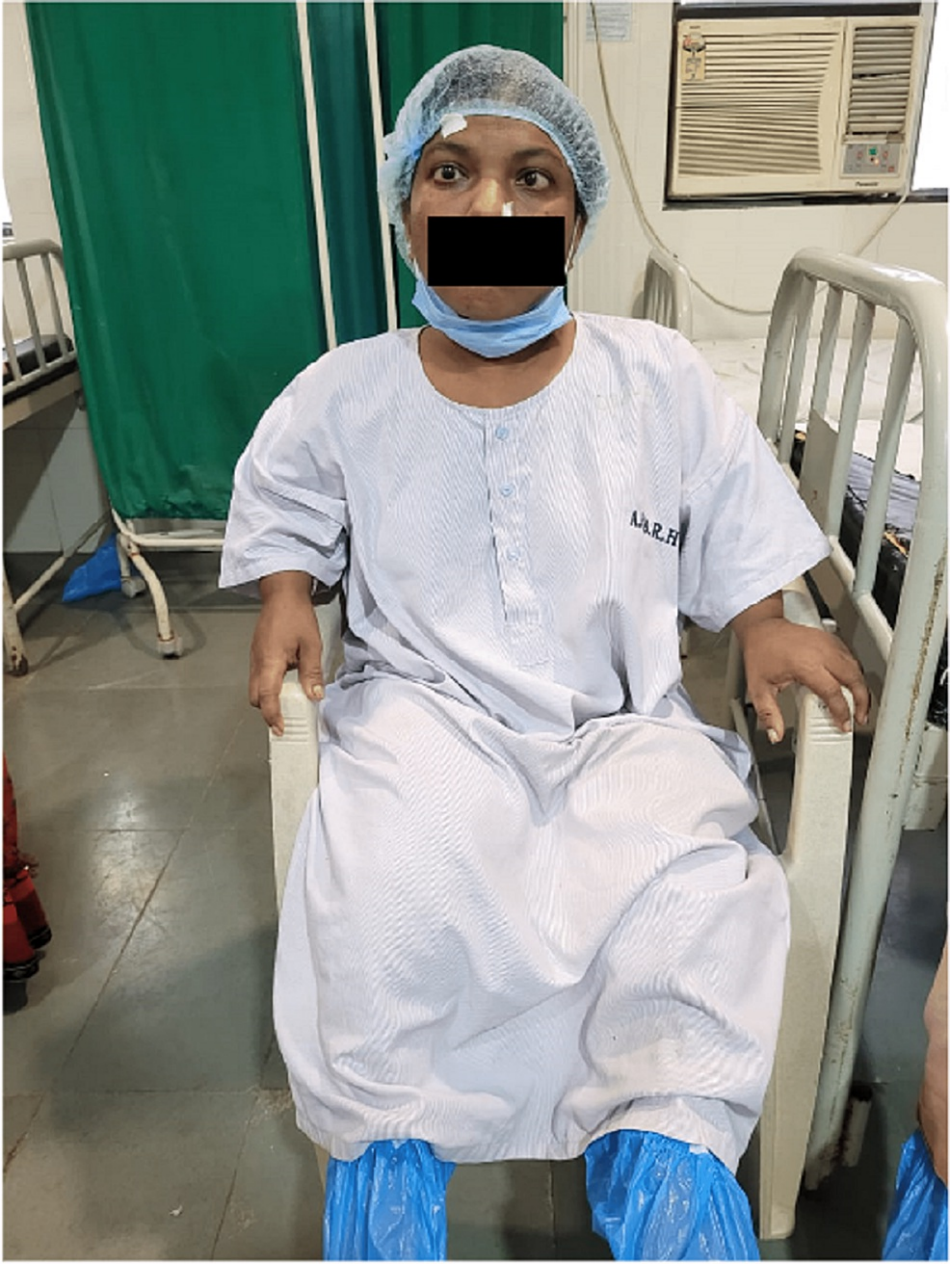
Clinical appearance of achondroplasia

A 25-year-old female presented to the Ophthalmology outpatient department (OPD) complaining of diminished vision in both eyes. She also complained of deviation of the left eye since birth. On examination, she had short stature, a distorted skull, short rhizomelic limbs, and increased spinal curvature. Her cognitive and auditory functions were normal. An orthopedic opinion was taken, and the patient was found to have achondroplasia (Figure 1).

**Figure 2:**
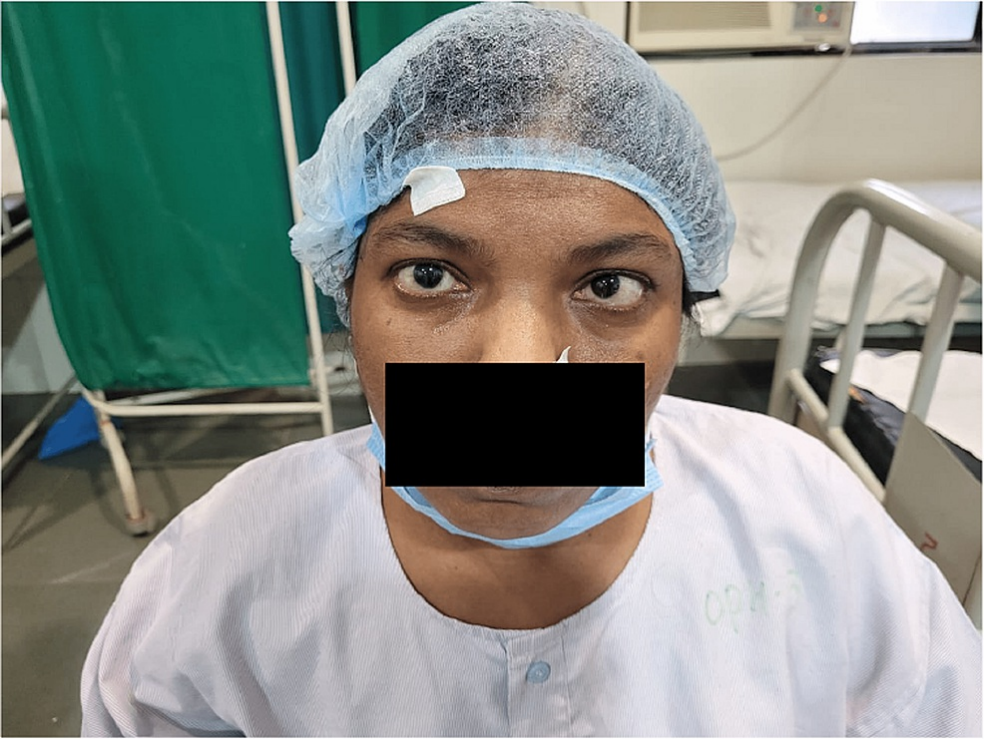
Esotropia of 30 degrees in the left eye

On ophthalmological examination, the visual acuity in the right eye was 6/36, improving to 6/24 on the pinhole, and that in the left eye was 6/24, improving to 6/18 on the pinhole. On torch light examination, the patient had an immature cataract in both eyes with esotropia of 30 degrees in the left eye (Figure 2).

**Figure 3:**
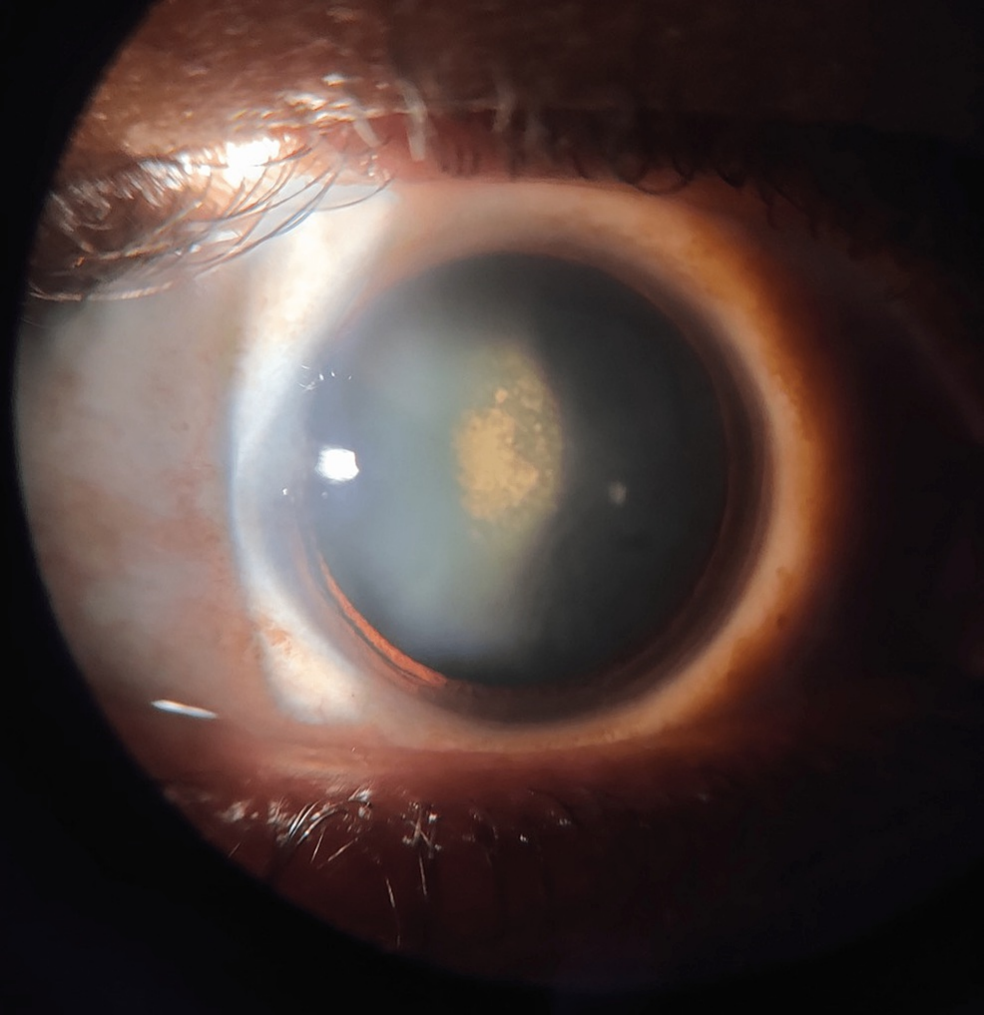
Posterior subcapsular and posterior polar cataract in the right eye

**Figure 4:**
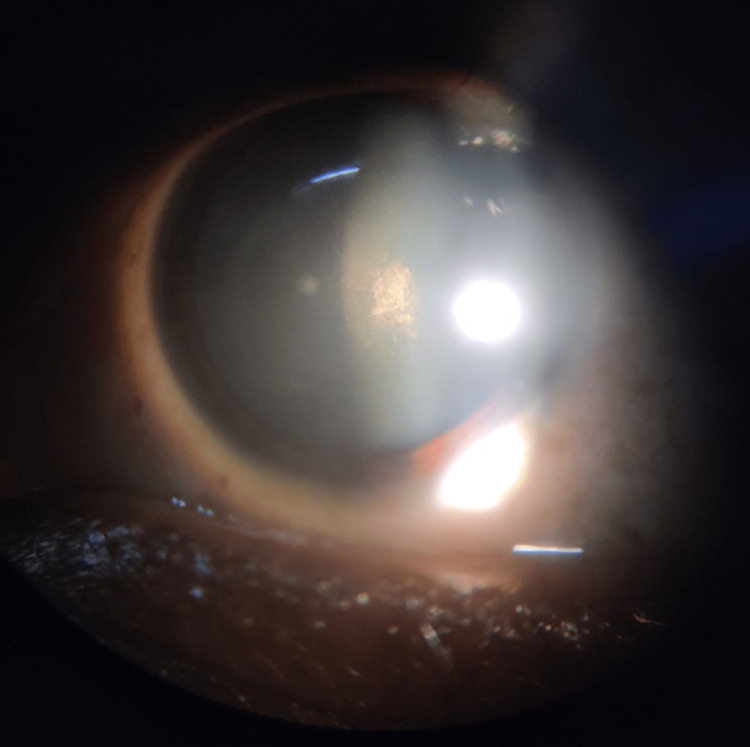
Posterior subcapsular and posterior polar cataract in the left eye

A slit-lamp examination showed a posterior subcapsular cataract with a posterior polar cataract in both eyes (Figures 3, 4).

**Figure 5:**
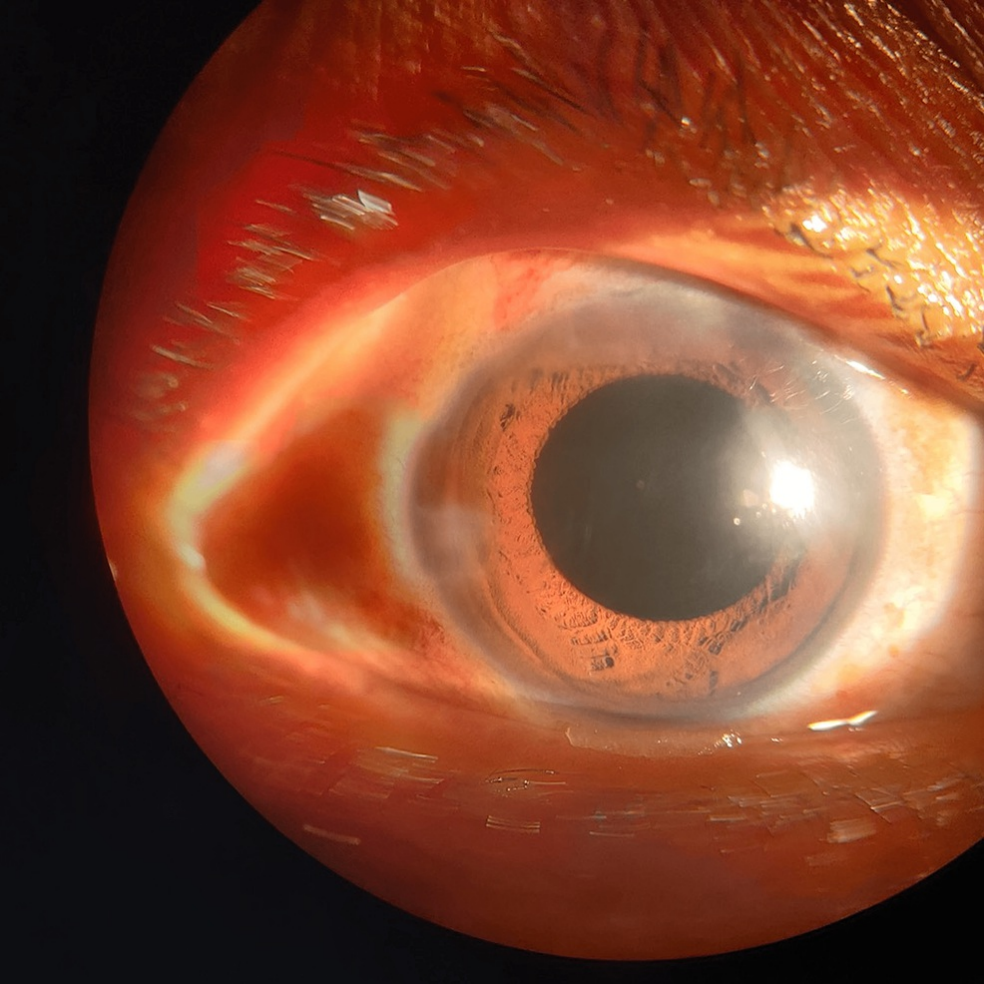
Postoperative day 1

The patient was operated on in the right eye with cataract extraction by phacoemulsification. Special care was taken due to the posterior polar cataract component. A dispersive viscoelastic substance was used, a larger capsulorhexis was done, and hydrodelineation was done instead of hydrodissection. The phaco machine parameter settings were changed and kept at a lower energy level with a lower aspiration flow rate and bottle height. A rigid PMMA 5-mm posterior chamber intraocular lens (PCIOL) was implanted into the bag. The postoperative visual prognosis was excellent, showing a visual acuity of 6/9 (Figure 5).

## Discussion

Achondroplasia is caused by pathogenic mutations in the FGFR3 and fibroblast growth factor genes. Rosenthal et al. [5] have described the ophthalmologic characteristics of patients with achondroplasia. They reported that of the 52 patients, 26 (50%) had telecanthus, and 10 (20%) had bilateral inferior oblique muscle overactivity and V-type exotropia. Five patients presented with tortuous retinal blood vessels. Angle abnormalities, such as the definite existence of the iris process, incomplete sequestration, and aberrant tissue at the anterior angle, were present in 26 of the 46 cases. Children with achondroplasia were found to have both Duane retraction syndrome and cone-rod retinal degeneration, according to Guirgis et al. [6]. According to Garg et al. [7], children with achondroplasia were found to have fundus albipunctatus in some cases.

Achondroplasia is caused by a single gene's genetic abnormalities, as reported by Maumenee and Mitchell [8]. The disease occurs in the early stages of the development of life, and these authors found a high incidence of systemic and central organ malformation. Achondroplasia and developing cataracts have not previously been positively linked, according to reports. Sharma et al. [9] have reported an association between the pseudo-achondroplastic variant of multiple epiphyseal dysplasia with cataracts and vitiligo. While the likelihood of a genetic connection is increased by the coexistence of developmental cataracts and achondroplasia, a chance association cannot be ruled out either. About three out of every 1,000 live births have developmental cataracts. Genetic mutations, typically autosomal dominant, are the most frequent cause, but other factors, such as chromosomal abnormalities, metabolic diseases, and prenatal infections, can also contribute. Additionally, skeletal abnormalities, including Hallerman-Streiff-François syndrome and Nance-Horan syndrome, have been linked to developmental cataracts. As a result, the skeletal ailment achondroplasia, which also affects development, can be linked to developmental cataracts. Patil et al. [10] have reported a pediatric case showing the association between achondroplasia and bilateral developmental cataract. The authors have highlighted the importance of early diagnosis and treatment of cataracts in patients with achondroplasia.

## Conclusions

We presented a case of achondroplasia with bilateral developmental cataracts. This syndrome has been largely ignored and patients rarely undergo ophthalmological examination. The patients are usually of a younger age group and may present at a later stage with severe visual impairment. Hence, screening all achondroplasia patients for cataracts and other ophthalmological anomalies is very crucial for timely intervention and management.
